# Trueness and precision of smartphone-based 3D scan in the assessment of facial edema in orthognathic surgery

**DOI:** 10.1016/j.jobcr.2026.101467

**Published:** 2026-05-27

**Authors:** Allan Vinícius Martins-de-Barros, Alysson Nunes de Lacerda, Leonardo Dias Pionório, Letícia Francine Silva Ramos, Tatiane Fonseca Faro, Bergson Carvalho de Moraes, Fernanda Souto Maior dos Santos Araújo, Gustavo Pina Godoy, Fábio Andrey da Costa Araújo

**Affiliations:** a Universidade de Pernambuco, Multicampi Garanhuns, Programa de Pós-Graduação em Saúde e Desenvolvimento Socioambiental, Arcoverde, Pernambuco, Brazil; b Universidade de Pernambuco, Campus Santo Amaro, Programa de Pós-Graduação em Ciências da Saúde, Recife, Pernambuco, Brazil; c Universidade de Pernambuco, Campus Santo Amaro, Programa de Pós-Graduação em Odontologia, Recife, Pernambuco, Brazil; d Universidade Federal de Pernambuco, Programa de Pós-Graduação em Odontologia, Recife, Pernambuco, Brazil; e Universidade de São Paulo, College of Dentistry, Postgraduate Program in Dental Sciences, São Paulo, São Paulo, Brazil; f Universidade de Pernambuco, Campus Santo Amaro, School of Dentistry, Recife, Pernambuco, Brazil

**Keywords:** Orthognathic surgical procedures, Photogrammetry, 3D facial scanning, Dimensional measurement accuracy

## Abstract

**Background:**

Although smartphone-based 3D facial scanning represents a low-cost tool for evaluating edema in maxillofacial surgery, evidence regarding its accuracy for clinical applications remains scarce. This study aimed to evaluate the trueness and precision of Qlone3D Scanner for smartphone in assessing postoperative edema following orthognathic surgery.

**Methods:**

This is a trueness and precision study. 3D facial reconstruction of computed tomography (CT) scans served as standard-reference. Both 3D facial models were converted to.STL format and imported to CloudCompare 2.14 for comparative mesh-to-mesh analysis. Trueness was defined by the proximity of smartphone-based scans to CT scans, while precision was assessed through comparison of subsequent smartphone-based scans. Statistical analysis was performed with SPSS 20.0.

**Results:**

Twenty-one participants were included. Trueness analysis showed similar average distance between mesh points in both preoperative (2.03 ± 0.74 mm) and postoperative (2.00 ± 0.68 mm). Precision analysis showed variability in the average distance between mesh points, with a mean of 2.97 ± 3.15 mm in preoperative period and of 1.89 ± 1.51 mm in postoperative period. No statistically significant differences were observed between preoperative and postoperative trueness or precision measurements (p > 0.05). The lower third of the face exhibited the greatest areas of divergence between mesh points.

**Conclusion:**

Postoperative facial edema did not significantly affect the trueness or precision of the Qlone3D Scanner. However, limitations in accurate surface reproduction were identified, restricting its applicability primarily to edema monitoring and underscoring the need for further refinement to ensure reliable clinical and research use.

## 1 Introduction

Orthognathic surgery aims to correct irregularities in the position of the jaws and teeth.^1^ Postoperative edema is a common and anticipated response following orthognathic surgery, arising as part of the acute inflammatory process triggered by surgical trauma. This physiological reaction involves increased vascular permeability mediated by inflammatory cytokines, leading to the extravasation of plasma proteins and fluid into the interstitial space. Clinically, it manifests as facial swelling, which is most evident within the first 48 to 72 h after the procedure and may persist for months postoperatively.^2^

Various methods have been proposed to quantify postoperative edema in oral and maxillofacial surgeries, ranging from direct measurements to more complex imaging examinations, such as ultrasound and computed tomography, which may not always be accessible or suitable for routine clinical practice. In this context, three-dimensional (3D) facial scanning has been suggested as a non-invasive and effective alternative^2^, ^3^, ^4^, ^5^.

Several technologies are available for obtaining 3D facial models, with conventional stereophotogrammetry systems being one of the most widely used. These systems employ synchronized cameras to capture multiple images of the face from different angles, reconstructing spatial coordinates through dedicated algorithms to generate three-dimensional facial models from two-dimensional images^6^, ^7^, ^8^.

More recently, the development of smartphone-based 3D facial scanning applications, which are also based on stereophotogrammetric principles, has emerged as an innovative alternative to conventional facial scanning systems. In addition to being accessible and cost-effective, smartphone-based 3D facial scanning offers speed, simplicity, and rapid generation of detailed facial models, expanding its potential applicability in different areas of dentistry.^2^^,^^4^^,^^5^

Although smartphone-based 3D facial scanning has been previously applied in clinical contexts, evidence regarding its accuracy for postoperative assessment remains limited.^9^ Evaluation of trueness and precision is particularly relevant because postoperative edema alters facial soft tissues and may affect image acquisition and facial reconstruction algorithms, potentially influencing the accuracy of facial models generated by stereophotogrammetry-based softwares.^10^

Therefore, this study aims to evaluate the trueness and precision of smartphone-based 3D facial scanning as a tool for assessing postoperative facial edema in individuals undergoing orthognathic surgery.

## 2 Materials and methods

### 2.1 Study design

This is a prospective study of trueness and precision. The index test under evaluation was smartphone-based 3D facial scanning using the application Qlone 3D Scanner (Eyecue Vision Technologies Ltd, Yokne'am Illit, IL), while multislice computed tomography (CT) served as reference test and current clinical reference standard for 3D assessment of facial soft tissues in orthognathic surgery. The target condition of the evaluation was the assessment of facial surface in individuals undergoing orthognathic surgery.

The Research Ethics Committee of the University of Pernambuco approved the study protocol under approval number 6.823.460 (CAAE: 75893423.9.0000.5207). All procedures conducted in this study were in line with the ethical standards of the institutional and national research committee, as well as the Declaration of Helsinki and its subsequent amendments or equivalent. Participants in the study signed the Informed Consent to formalize their participation in the study. This observational study adhered to the STROBE guidelines to ensure methodological rigor and transparent reporting of results.

### 2.2 Study population and sample

The study population comprised individuals treated at Oswaldo Cruz University Hospital (HUOC) who were subjected to orthognathic surgery for correction of dentofacial deformities. Inclusion criteria were individuals without comorbidities (ASA I, according to the American Society of Anesthesiology classification); aged over 18 years; both sexes; with a confirmed indication for orthognathic surgery based on a clinical diagnosis issued by an Oral and Maxillofacial Surgeon from the service. Participants who were chronically using medications that affect postoperative edema, those who did not attend the first postoperative follow-up appointment, those who did not undergo control CT scanning on 4th postoperative day, or those who withdrew from the study at any point were excluded from the research.

Sample size estimation was based on a pilot study including 10 participants, conducted under the same acquisition protocol as the main study. The maximum distance between trueness mesh points was used as the reference variable to estimate the expected mean difference between preoperative (mean: 18.18 ± 7.21) and postoperative (mean: 12.29 ± 5.93) measurements. Assuming a paired 1:1 design, 80% statistical power, and a 95% confidence level, a minimum of 20 participants was required. To account for potential losses, the sample size was increased by 25%, resulting in a final sample of 25 participants. A total of six smartphone-based 3D facial scanning (three preoperative and three postoperative) and two facial CT scans (one preoperative and one postoperative) were obtained from each participant.

### 2.3 Demographic and clinical data collection

After signing Informed Consent, eligible participants' sociodemographic (age, sex, and skin color) and clinical data (dentofacial deformity and surgical intervention) were collected based on hospital records.

### 2.4 Acquisition of computed tomography images

Facial computed tomography images were obtained during preoperative planning and postoperative follow-up of the patient. Volumetric image acquisition in the axial plane was performed using a multislice multidetector CT scanner (Toshiba X-Vision) without contrast media, with the participant in a horizontal supine position. Image files obtained by CT scanner in DICOM (Digital Imaging and Communications in Medicine) format were imported into InVesalius software (version 3.1.1), where multiplanar 3D reconstruction of soft tissues of the face was performed, generating a file in Standard Triangle Language (STL) format.

CT imaging was selected as a reference modality due to its routine clinical use in orthognathic surgery, standardized acquisition protocol, and high spatial resolution, allowing reproducible 3D reconstruction of facial soft tissues without additional imaging burden or radiation exposure beyond standard care.

### 2.5 Acquisition of smartphone-based 3D facial scans

Smartphone-based 3D facial scan images of each participant were obtained using an iPhone 12 smartphone with Qlone 3D Scanner application. The participant's position during facial scanning was standardized: they were seated on a height-adjustable swivel chair at a fixed distance from the scanner, maintaining an upright posture with their hands resting on their lap. The midline of the face was aligned toward the camera and participants were instructed to look at a fixed reference point positioned on the wall in front of them at eye level while maintaining a neutral facial expression.

Participants underwent facial scanning in the preoperative period, one week before surgery, and at the first follow-up visit on 4th postoperative day. At each time point, three consecutive facial scans were performed for precision analysis, obtaining three 3D facial models in STL format.

### 2.6 Superimposition of 3D meshes for trueness and precision measurements

The STL files of 3D reconstruction of soft tissues from CT scans and 3D scanned facial models were imported into Meshmixer software (Autodesk®, Inc., San Rafael, CA, USA) for preprocessing and delimitation of the Facial Area of Interest (FAI). The FAI included the facial area bounded superiorly by the glabella, inferiorly by the gnathion and laterally by the right and left tragus.^7^

Following FAI segmentation, the meshes were imported into CloudCompare software (version 2.14 Unified) for 3D superimposition and comparative analysis. Initial alignment was performed using five reproducible anatomical landmarks (nasion, pogonion, pronasale, and bilateral zygomatic prominences), which were consistently identified on all meshes by a single trained operator. This landmark-based alignment was followed by fine registration using CloudCompare's alignment tool, allowing point-to-point distance calculation between a Reference File (RF) and a Compared File (CF). Point-to-point distances were calculated across all mesh vertices within the predefined FAI. The software generated a Gaussian-distributed histogram representing the spatial deviation between corresponding mesh points. The measures generated from the superimpositions between RF and CF were used for trueness and precision analysis.

For this analysis, trueness was defined as the degree of agreement between the smartphone-based 3D facial scan and the reference CT-derived facial model. Accordingly, for each time point (preoperative and postoperative), the CT-derived mesh was used as the RF, while the first facial scan of the corresponding time point served as the CF.

Precision was defined as the repeatability of the smartphone-based scanning method under identical conditions. For each time point (preoperative and postoperative), three consecutive facial scans were sequentially acquired by the same operator during a single clinical session, within a short time interval (<5 min) between acquisitions. The procedure was performed under standardized conditions with participants instructed to maintain a neutral facial expression and head position throughout the acquisition. The first scan was used as the RF, and the two subsequent scans were used as CFs for precision analysis.

From each superimposition, quantitative metrics were extracted, including mean point-to-point distance, standard deviation of distances, and maximum and minimum deviation values. Measurements closer to zero indicated higher trueness or precision, whereas increasing deviation values reflected reduced agreement or repeatability.

It is important to note that discrepancies between CT-derived meshes and surface scans may partly reflect methodological differences, including patient positioning (supine vs. upright) and variations in mesh density, rather than solely scanner inaccuracy.

### 2.7 Statistical analysis

The database was built using SPSS® software, version 20.0.0. The data were analyzed descriptively, with categorical variables presented as absolute and relative frequencies, and quantitative variables presented using measures of central tendency (mean and median) and dispersion (standard deviation).

Inferential statistical analysis was conducted to assess whether presence of postoperative edema significantly affected trueness and precision of facial scanning compared to preoperative. Kolmogorov-Smirnov test was used to test data normality. Variables with a normal distribution were analyzed using paired *t*-test, while variables with a non-normal distribution were analyzed using Wilcoxon test. A significance level of 95% (p < 0.05) was adopted for all tests.

## 3 Results

**Supplementary Fig. 1:**
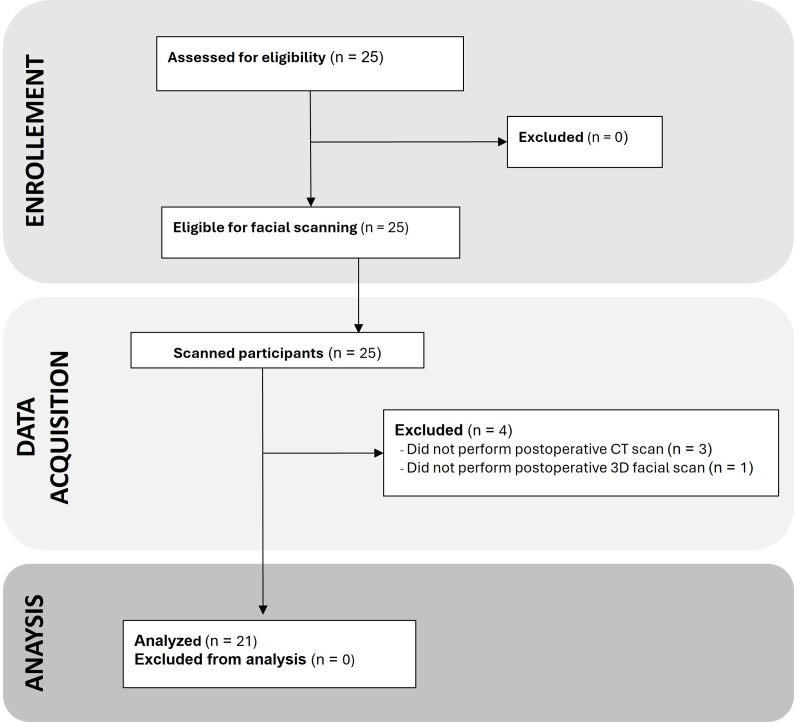
Flowchart of the participants of the study.

Twenty-five individuals were initially eligible for the study. However, four were excluded due to failure to attend postoperative CT or smartphone-based 3D facial scans. Thus, 21 participants completed all stages and were included in the final analysis of the study (Supplementary Fig. 1).

The age range of the individuals included in the study varied from 20 to 42 years, with a mean age of 28.9 ± 5.7 years. Women (76.2%) and individuals of mixed race (52.4%) comprised the majority of the sample. A predominance of individuals with skeletal class III facial profile (57.1%) was observed compared to those with skeletal class II (42.9%). Regarding the surgical intervention, almost all participants (95.2%, n = 20) were subjected to combined maxillary and mandibular surgery, while 57.1% (n = 12) also were subjected to genioplasty. Only one participant was subjected to isolated mandibular surgery. Detailed sociodemographic and clinical data are presented in Table 1.

**Table 1:** Baseline sociodemographic and clinical characteristics of the study participants.

**Age**	**Years**	

Age range	20 – 42	
Mean ± SD	28.9 ± 5.72	

**Sex**	**n**	**%**

Women	16	76.2
Men	05	23.8

**Skin color**	**n**	**%**

Brown	11	52.4
White	08	38.1
Black	02	9.5

**Dentofacial deformity**	**n**	**%**

Skeletal class II	09	42.9
Skeletal class III	12	57.1

**Surgical intervention**	**n**	**%**

Maxilla (Le Fort I osteotomy)	20	95.2
Mandbile (Bilateral sagital split osteotomy)	21	100.0
Chin (Genioplasty)	12	57.1

**Fig. 1:**
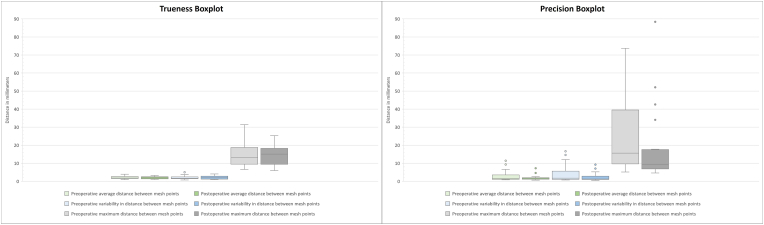
Boxplot of trueness and precision comparisons.

Trueness and precision data for smartphone-based 3D facial scans in preoperative (without edema) and postoperative (with edema) periods are shown in Table 2 and illustrated in boxplots in Fig. 1.

**Table 2:** Trueness and precision measurements for 3D facial scans obtained with Qlone 3D Scanner in preoperative (without edema) and postoperative (with edema) periods.

Trueness measures	Range (Min. – Max.)	Mean ± SD	Median	*p* Value*
Preoperative average distance between mesh points	0.97 – 3.92	2.03 ± 0.74	1.88	0.900
Postoperative average distance between mesh points	1.04 – 3.28	2.00 ± 0.68	1.93	
Preoperative variability in distance between mesh points	0.77 – 5.55	2.03 ± 0.99	1.65	0.979
Postoperative variability in distance between mesh points	0.94 – 4.12	2.04 ± 0.89	1.94	
Preoperative maximum distance between mesh points	6.57 – 31.37	14.77 ± 6.47	13.23	0.698
Postoperative maximum distance between mesh points	5.97 – 25.26	14.40 ± 5.54	15.05	

Precision measures	Range (Min. – Max.)	Mean ± SD	Median	*p* Value**
Preoperative average distance between mesh points	0.78 – 11.82	2.97 ± 3.15	1.58	0.244
Postoperative average distance between mesh points	0.82 – 7.39	1.89 ± 1.51	1.35	
Preoperative variability in distance between mesh points	0.75 – 16.72	4.19 ± 5.10	1.72	0.181
Postoperative variability in distance between mesh points	0.67 – 9.33	2.48 ± 2.50	1.18	
Preoperative maximum distance between mesh points	5.14 – 73.74	25.36 ± 22.36	15.65	0.079
Postoperative maximum distance between mesh points	4.63 – 88.40	17.75 ± 20.66	9.44	

Min., minimum. Max., maximum. SD, standard deviation. *Paired *t*-test. **Wilcoxon test.

Trueness analysis showed that the average distance between mesh points were similar in both preoperative (mean = 2.03 ± 0.74 mm) and postoperative (mean = 2.00 ± 0.68 mm) comparisons. Furthermore, the mean variability of the distance between points was 2.09 ± 0.98 mm in preoperative and 2.12 ± 0.69 mm in postoperative comparisons. In the precision analysis, the average distance between mesh points of each superimposition ranged from 0.78 to 11.82 mm with a mean of 2.97 ± 3.15 mm in preoperative period, and from 0.82 to 7.39 mm with a mean of 1.89 ± 1.51 mm in postoperative period. The mean variability of the distance between points in preoperative and postoperative periods was 4.19 ± 5.10 mm and 2.48 ± 2.50 mm, respectively.

The maximum distance between points of superimposed meshes from subsequent 3D facial scans was higher than in trueness analysis, reaching 73.74 mm in preoperative and 88.40 mm in postoperative comparisons. However, extreme maximum deviation values likely represent localized artifacts or edge mismatches during mesh alignment, rather than clinically meaningful global distortion. In both trueness and precision analyses, the minimum distance between the points of the superimposed meshes was 0 mm in all comparisons.

No statistically significant differences were observed between preoperative and postoperative trueness or precision measurements (p > 0.05).

**Fig. 2:**
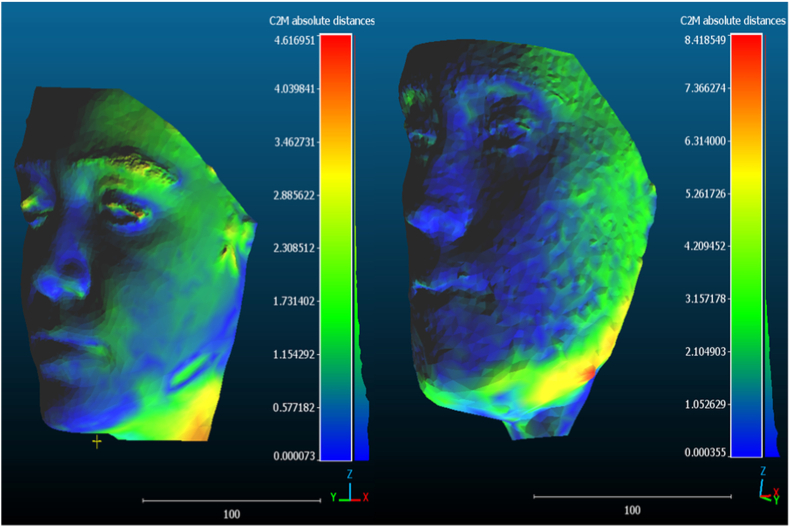
Colormaps with areas of point dispersion in mesh superimpositions in trueness analysis (left) and precision analysis (right). Warmer colors indicate greater point-to-point deviation, while cooler colors represent smaller discrepancies between superimposed meshes. The greatest areas of divergence were consistently observed in the lower third of the face, particularly in the submandibular, submental, and perioral regions. Mesh comparisons and color maps were generated using CloudCompare software (version 2.14 Unified).

In qualitative analysis of the meshes’ superimpositions, the lower third of the face exhibited the greatest areas of divergence between mesh points, including submandibular, submental and perioral regions. Orbital and supraorbital regions also showed evidence of point dispersion, though less frequently. The same distortion pattern was observed in both trueness and precision analyses (Fig. 2).

**Fig. 3:**
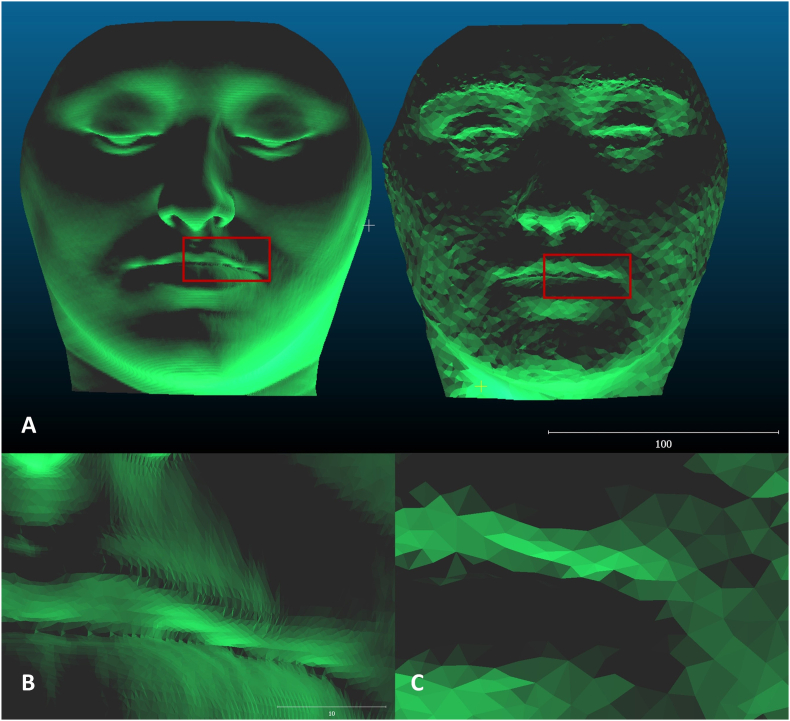
Comparison of mesh resolution between imaging modalities. **A,** STL meshes showing the difference in resolution between the 3D soft tissue reconstruction from a CT scan (left) and the 3D stereophotogrammetry facial scan generated with Qlone 3D Scanner (right). **B,** Higher magnification of the 3D soft tissue reconstruction from the CT scan mesh. **C,** Higher magnification of the 3D stereophotogrammetry facial scan mesh.

Additionally, STL-converted meshes obtained from CT scans exhibited a higher density of captured data compared to those obtained through smartphone-based 3D facial scanning, as shown in Fig. 3.

## 4 Discussion

The main advantages of smartphone-based 3D facial scanning include rapid acquisition, low cost, integrated processing systems, ease of use, and non-invasive characteristics. Several smartphone-based 3D facial scanning systems have been proposed and evaluated in the literature; however, some have been discontinued or present limitations related to availability and performance^11^, ^12^, ^13^, ^14^. Although previous studies have assessed trueness and precision of smartphone-based 3D facial scanning systems, the present investigation specifically explored the potential influence of postoperative facial edema on these parameters, using a clinically applicable acquisition protocol.^15^

Aung et al.^16^ defined reliability intervals for trueness and precision measurements, classifying them as highly reliable (0–1 mm), reliable (1–1.5 mm), moderately reliable (1.5–2 mm), and unreliable (>2 mm). In the present study, mean trueness and precision values exceeded 2 mm in both preoperative and postoperative assessments, placing them within the unreliable range according to these criteria. Similar observations were reported by D'Ettorre et al.*,*^12^ who considered deviations greater than 1 mm as clinically relevant, reinforcing the limited absolute accuracy of the smartphone-based system evaluated.

Despite the lack of statistically significant differences between scans acquired with and without postoperative edema, the magnitude of the observed deviations indicates that this technology may not be suitable for applications requiring high geometric fidelity, such as detailed volumetric or morphometric analyses.

However, the consistency of measurements across conditions suggests potential applicability for longitudinal monitoring of facial edema trends, where relative changes over time are of greater clinical interest than absolute dimensional accuracy. Considering that postoperative facial edema can exceed the magnitude of the observed deviations, these findings support the potential use of this technology for postoperative monitoring, where relative changes over time may remain detectable despite limitations in absolute trueness or precision.

These findings may be partially explained by the intrinsic difficulty in maintaining a completely static facial posture throughout the scanning process. Minor involuntary movements involving the forehead, lips, mandible, or ocular region can alter the reconstructed surface geometry, negatively impacting both trueness and precision.^14^^,^^17^^,^^18^ Although sequential scans were acquired under standardized conditions within the same clinical session, subtle motion artifacts cannot be fully eliminated and remain an inherent limitation of surface-based facial scanning methods.

Cascos et al.*,*^17^ in a study with 60 participants, compared different facial metric acquisition techniques (manual, 2D, and 3D) in terms of trueness and precision. Their results showed an average trueness value of 0.61 mm (±1.65) for facial scanning, whereas the present study demonstrated mean deviations greater than 2 mm, underscoring the technical limitations of smartphone-based devices when compared to more robust, high-cost facial scanning technologies.

The use of conventional cameras as facial scanning sensors was first described in the literature in 2017, employing depth-sensing technologies capable of generating real-time depth data. More recently, smartphones incorporate light-based ranging technologies that calculate object distance through emitted and reflected light pulses, enabling 3D scene reconstruction.^19^ Nevertheless, when compared to dedicated stereophotogrammetry systems, smartphone sensors still generate lower-resolution meshes and are more susceptible to noise, artifacts, and surface discontinuities, which may compromise measurement accuracy.^13^^,^^20^

Gibelli et al.^20^ demonstrated that even high-end stereophotogrammetry systems show region-dependent variability, with optimal accuracy limited to areas under favorable visualization conditions. Consistent with those findings, the lower third of the face exhibited the greatest deviations in the present study, a region particularly susceptible to motion and soft-tissue deformation.

It is important to consider that the meshes generated by facial scans are subject to interference from external factors such as lighting conditions, shadowing, involuntary facial movements, and variations in image capture angles. These elements introduce a degree of variability that reflects limitations inherent to study methods.^14^^,^^17^^,^^20^ Additionally, CT-derived facial models present higher polygon density and spatial resolution than surface scans, which may accentuate discrepancies during superimposition. Differences in patient positioning during image acquisition (supine for CT and upright for facial scanning) may also influence soft-tissue morphology and partially account for the observed deviations, potentially leading to an underestimation of trueness in this study.^13^

Optimal facial area coverage in stereophotogrammetry systems is crucial for ensuring trueness and precision in morphometric measurements, particularly in critical areas such as subnasal region, lips, jaw, eyes, and hair-covered regions, where capture failures are frequently reported in the literature.^12^^,^^14^ These findings align with the results of this study. Although a standardized scanning protocol was adopted,^14^ protocol standardization alone may not fully compensate for intrinsic hardware and algorithmic limitations. Future investigations should focus on protocol optimization and technological refinements to improve reproducibility and clinical reliability.

Furthermore, the manipulation of facial tissues during orthognathic surgery leads to changes in shape, contour and surface, caused not only by the repositioning of bone structures but also by local edema.^2^^,^^21^ Importantly, variability in postoperative edema may have been influenced by factors such as surgical duration, extent of skeletal movement, intraoperative bleeding, and surgeon-related variables, which were not controlled in this study. However, as the primary objective was to assess scanner performance rather than edema severity, these factors are unlikely to have significantly impacted the main outcomes.

Additionally, the present findings indicate that postoperative facial edema did not significantly affect trueness or precision values, suggesting that edema itself does not represent an additional limiting factor for smartphone-based 3D facial scanning. These findings should be interpreted within the context of early postoperative assessment (fourth postoperative day) and predominantly bimaxillary surgical procedures, which may limit their generalizability to other time points or surgical approaches. Nonetheless, given the overall magnitude of deviations observed, results should be interpreted with caution, particularly when extrapolating to precise volumetric assessments.

## 5 Conclusion

The results of this study suggest that edema does not seem to interfere with the trueness and precision of smartphone-based 3D facial scanning. However, the magnitude of the observed deviations indicates that this technology currently presents limited absolute accuracy, restricting its use for detailed or absolute measurements in facial surface. Despite these limitations, the consistency of measurements across preoperative and postoperative conditions suggests that smartphone-based 3D facial scanning may be suitable for longitudinal monitoring of facial edema trends, where relative changes over time are clinically relevant. Further technological improvements in image acquisition and reconstruction algorithms, as well as validation in larger samples, are required before broader clinical application can be recommended.

## Human subjects statement

All procedures involving human participants were conducted in accordance with the ethical standards of the institutional and/or national research committee and with the 1964 Helsinki Declaration and its subsequent amendments.

## Informed consent statement

Informed consent was obtained from all subjects involved in the study.

## Ethical clearance

The Research Ethics Committee of the University of Pernambuco approved the study protocol under approval number 6.823.460 (CAAE: 75893423.9.0000.5207).

## Institutional review board statement

The study was conducted in accordance with the Declaration of Helsinki, and approved by the Institutional Research Ethics Committee of the University of Pernambuco (Approval number 6.823.460/CAAE: 75893423.9.0000.5207).

## Funding

None.

## Declaration of competing interest

The authors declare that they have no known competing financial interests or personal relationships that could have appeared to influence the work reported in this paper.

## References

[1] Olejnik A., Verstraete L., Croonenborghs T.-M., Politis C., Swennen G.R.J. (2024). The accuracy of three-dimensional soft tissue simulation in orthognathic Surgery-A systematic review. J Imaging.

[2] Buitenhuis M.B., Klijn R.J., Rosenberg A.J.W.P., Speksnijder C.M. (2022). Reliability of 3D stereophotogrammetry for measuring postoperative facial swelling. J Clin Med.

[3] Kau C.H., Cronin A.J., Durning P., Zhurov A.I., Sandham A., Richmond S. (2006). A new method for the 3D measurement of postoperative swelling following orthognathic surgery. Orthod Craniofac Res.

[4] Oliveira Z.S.B., Silveira M.L.M., Gomes P.P., Silva J.S.P., Germano A.R. (2021). Early recovery after surgery protocol in orthognathic surgery: a randomized, blind clinical study. Braz Oral Res.

[5] Friscia M., Seidita F., Committeri U. (2022). Efficacy of Hilotherapy face mask in improving the trend of edema after orthognathic surgery: a 3D analysis of the face using a facial scan app for iPhone. Oral Maxillofac Surg.

[6] Abdel-Alim T., Iping R., Wolvius E.B. (2021). Three-dimensional stereophotogrammetry in the evaluation of craniosynostosis: current and potential use cases. J Craniofac Surg.

[7] Cappella A., Solazzo R., Gigante L. (2024). Comparison of different 3D surface registration-based methods to assess facial asymmetry. Diagnostics (Basel).

[8] Andrews J., Alwafi A., Bichu Y.M., Pliska B.T., Mostafa N., Zou B. (2023). Validation of three-dimensional facial imaging captured with smartphone-based photogrammetry application in comparison to stereophotogrammetry system. Heliyon.

[9] Caputo A.A., Rubino E., Marcianò A. (2023). Three-dimensional facial swelling evaluation of piezo-electric vs conventional drilling bur surgery of impacted lower third molar: a randomized clinical trial. BMC Oral Health.

[10] Dallazen E., Baccaro G.C., Santos A.M.S. (2023). Comparison of manual (2D) and digital (3D) methods in the assessment of simulated facial edema. J Oral Maxillofac Surg.

[11] Rudy H.L., Wake N., Yee J., Garfein E.S., Tepper O.M. (2020). Three-dimensional facial scanning at the fingertips of patients and surgeons: accuracy and precision testing of iPhone X three-dimensional scanner. Plast Reconstr Surg.

[12] D'Ettorre G., Farronato M., Candida E., Quinzi V., Grippaudo C. (2022). A comparison between stereophotogrammetry and smartphone structured light technology for three-dimensional face scanning. Angle Orthod.

[13] Thurzo A., Strunga M., Havlínová R. (2022). Smartphone-based facial scanning as a viable tool for facially driven orthodontics?. Sensors.

[14] Heike C.L., Upson K., Stuhaug E., Weinberg S.M. (2010). 3D digital stereophotogrammetry: a practical guide to facial image acquisition. Head Face Med.

[15] Verhoeven T.J., Vinayagalingam S., Claeys G., Xi T., Berge S.J., Maal T.J.J. (2024). Does facial asymmetry vary between subjects of different age groups? A 3D stereophotogrammetry analysis. J Craniomaxillofac Surg.

[16] Aung S.C., Ngim R.C.K., Lee S.H. (1995). Evaluation of the laser scanner as a surface measuring tool and its accuracy compared with direct facial anthropometric measurements. Br J Plast Surg.

[17] Cascos R., Ortiz L., Álvarez-Guzmán F. (2023). Accuracy between 2D photography and dual-structured light 3D facial scanner for facial anthropometry: a clinical study. J Clin Med.

[18] Brons S., Darroudi A., Nada R. (2018). Influence of involuntary facial expressions on reproducibility of 3D stereophotogrammetry in children with and without complete unilateral cleft lip and palate from 3 to 18 months of age. Clin Oral Invest.

[19] Stevenson S., Liscio E. (2024). Assessing iPhone LiDAR & Recon‐3D for determining area of origin in bloodstain pattern analysis. J Forensic Sci.

[20] Gibelli D., Palamenghi A., Poppa P., Sforza C., Cattaneo C., Angelis Danilo De (2022). 3D‐3D facial registration method applied to personal identification: does it work with limited portions of faces? An experiment in ideal conditions. J Forensic Sci.

[21] Laureano Filho J.R., Oliveira e Silva ED., Camargo I.B., Gouveia F.M.V. (2005). The influence of cryotherapy on reduction of swelling, pain and trismus after third-molar extraction. JADA (J Am Dent Assoc).

